# Development of new liposomal formulations of quercetin – *in vitro* study

**DOI:** 10.1038/s41598-026-48068-3

**Published:** 2026-04-18

**Authors:** Klaudia Krawczyńska, Agnieszka Rusak, Tomasz Górnicki, Monika Mrozowska, Piotr Dzięgiel, Jerzy Gubernator

**Affiliations:** 1 Division of Histology and Embryology, Department of Human Morphology and Embryology, Wroclaw Medical University, Tytusa Chalubinskiego 6a St, Wroclaw, 50-368 Poland; 2 Department of Human Biology and Cosmetology, Faculty of Physiotherapy, Wroclaw University of Health and Sport Sciences, Ignacego Jana Paderewskiego 35, Wroclaw, 51-612 Poland; 3 Department of Lipids and Liposomes, Faculty of Biotechnology, University of Wrocław, Joliot-Curie 14a St, Wroclaw, 50-383 Poland

**Keywords:** Quercetin, Liposomes, Nanotechnology, Melanoma, Anticancer therapy, Biochemistry, Biotechnology, Cancer, Chemistry, Drug discovery

## Abstract

Quercetin is a chemical compound present in many fruits and vegetables, known for its antioxidant and anti-inflammatory effects. Additionally, this bioactive flavonoid can induce apoptosis in abnormally developing cells. The aim of the study was to develop an effective method for encapsulating quercetin in liposomes and to evaluate the drug encapsulation efficiency, stability of the formulation and anticancer effects on melanoma cells. The results showed that quercetin can be successfully encapsulated in the aqueous phase of liposomes by complexing it with copper ions. To assess the cytotoxicity of the liposomal quercetin, SRB assays were performed on two melanoma cell lines, A-375 and C32, as well as on normal human keratinocytes, HaCaT. This formulation exhibited a cytotoxic effect on melanotic melanoma cells but not on normal cells, indicating the possible use of liposomal quercetin in anticancer treatment.

**Supplementary Information:**

The online version contains supplementary material available at 10.1038/s41598-026-48068-3.

## Introduction

Malignant melanoma, which is the most dangerous type of skin cancer, results from the malignant transformation of melanocytes, melanin pigment-producing cells of neural crest origin^1^. The most common sites of metastasis are the nearby lymph nodes, but the cancer can also spread to distant organs such as the lungs, liver, and brain^2^. This epidemiological assessment of global cancer data estimated that in 2022 there were 331,647 new melanoma cases and 58,645 deaths due to melanoma^3^. If these rates remain stable, by 2040, the global burden of melanoma is estimated to increase to 510,000 new cases and 96,000 deaths^4^. However, the current trends show that despite the increasing number of cases, thanks to early diagnosis and improved access to modern therapies, the mortality rate of the disease has remained stable. Standard treatment options include surgery, but in advanced stages, when metastases are present, it is necessary to implement other methods such as chemotherapy, immunotherapy, or radiation therapy . Chemotherapy is a systemic treatment used to reduce symptoms caused by cancer, but it has a low response rate of 12.1–17.6% for dacarbazine (DTIC), the only U.S. Food and Drug Administration (FDA)-approved chemotherapy for melanoma^6^. Molecular targeted therapies, such as tyrosine kinase inhibitors (TKI), have been developed to target genetic biomarkers whose overexpression is implicated in tumorigenesis^7^. Approximately 50% of patients with melanoma have a mutation in the gene that encodes a protein called B-Raf (BRAF, B-Raf proto-oncogene, serine/threonine kinase), which promotes melanomagenesis through the constitutive activation of the mitogen-activated protein kinase (MAPK) pathway, leading to increased cell proliferation^8^. A BRAF mutation leads to dysregulation of this pathway, resulting in uncontrolled cell proliferation and tumor growth. Drugs currently used in therapy, such as dabrafenib and vemurafenib, are designed to block the dysfunctional BRAF kinase, thereby limiting the abnormally excessive cell proliferation^9, 10^. Other drugs, such as trametinib and cobimetinib, block mitogen-activated protein kinase (MEK) and thereby inhibit further signal transduction to mitogen-activated protein kinase 1 (ERK) in the same MAPK signaling pathway, and are also used in clinical practice^11^. BRAF kinase inhibitors and MEK kinase inhibitors are often used in combination to increase the effectiveness of the therapy^12, 13, 14, 15^.

Due to their prevalence and wide range of properties, including antioxidant, anti-inflammatory, and immunomodulatory activities, naturally occurring compounds in plants, particularly flavonoids, have received increasing research interest. Among this group of compounds is quercetin (Q), which may be protective against cancer through its antioxidant function in small doses, while in high doses, it acts as a pro-oxidant and can induce apoptosis in cancer cells^16^.

**Fig. 1:**
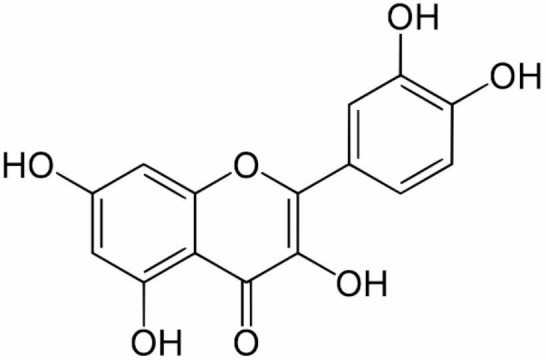
Chemical structure of Q.

The chemical structure of Q is characterized by the presence of three aromatic rings linked to several hydroxyl groups (Fig. 1). The phenolic hydroxyl groups of Q act as electron donors, and they are responsible for the radical-scavenging activity. Moreover, Q forms complex compounds with metal ions, such as copper, thereby preserving its biological activity^17, 18, 19^. According to the literature, Q has three potential metal-complexing domains that can interact with metal ions: 3′,4′-dihydroxy group in the B ring, as well as the 3-hydroxy or 5-hydroxy and the 4-carbonyl group in the C ring^20, 21^. Direct interactions of Q with copper ions were also confirmed by UV-VIS spectroscopy. The reaction resulted in the formation of a complex through the carbonyl oxygen and 3-hydroxy group in the C-ring^22^. Moreover, this complex has been shown to exhibit stronger radical-scavenging activity than Q alone^23^.

Previous studies have shown that Q, like the melanoma drugs discussed earlier, has the capability of targeting various molecular pathways such as MAPK, in cancer cells to suppress their proliferation^5, 24, 25^. Moreover, Q may also act synergistically with other compounds, enhancing their anticancer effects. One of the previous studies showed that Q may significantly improve response rates in tumors that express tyrosinase, including melanoma^26^. An important limitation of using Q in therapy is its relatively low bioavailability, therefore it is necessary to use biocompatible drug carriers, such as liposomes. These carriers have great potential in anticancer therapy, as they can be engineered to target cancer cells by adding ligands or antibodies that recognize cancer cell markers to their surface^27^. Due to its chemical structure and hydrophobic nature, Q can be encapsulated in nanoliposomes in two distinct ways: within the hydrophobic region of the lipid bilayer membrane or in the aqueous core, for example, through complexation with copper ions. Previous studies have described a method for preparing Q-loaded nanoliposomes using the lipid thin-film hydration technique with phosphate-buffered saline (PBS). In this approach, Q was primarily incorporated into the hydrophobic lipid bilayer^28^. Another study reported the encapsulation of Q in cyclodextrins, conventional liposomes, and drug-in-cyclodextrin-in-liposomes using the ethanol injection method with various types of phospholipids. The resulting Q-containing liposomes demonstrated physical stability after one year of storage at 4 °C^29^.

The aim of this study was to develop an efficient method for encapsulating Q in liposomes and to evaluate its cytotoxicity on two human melanoma cell lines, the malignant melanotic A-375 and the amelanotic C32, compared to the normal human keratinocyte line, HaCaT.

## Materials and methods

### Materials

Hydrogenated Soy Phosphatidylcholine (HSPC, purity ≥ 90.0%) and N-(carbonyl-methoxypolyethylene glycol-2000)−1,2-distearoyl-sn-glycero-3-phosphoethanolamine 2000 (DSPE-PEG2000) were purchased from Lipoid GmbH (Germany). Cholesterol (Chol) was purchased from Northern Lipids Inc. (Canada). Q (≥ 95%), copper (II) sulfate, trichloroacetic acid (TCA), acetic acid, sulforhodamine B (SRB) and Sephadex^®^ G-50 Fine were purchased from Sigma-Aldrich^®^ (USA). Chloroform, methanol and tert-butanol were purchased from Stanlab (Poland). Dimethylsulfoxide (DMSO), sodium chloride, ethylenediaminetetraacetic acid (EDTA) were purchased from ChemPur (Poland). Tris-HCl was purchased from Serva (Germany). Ultrapure water was obtained from Milli-Q^®^ system (Merck, Germany).

For cell cultures, Dulbecco’s Modified Eagle Medium (DMEM) was purchased from Capricorn (Germany). Minimum Essential Medium Eagle with Earle′s salts (EMEM) and PBS were purchased from Gibco^®^ (USA). Trypsin/EDTA solution, fetal bovine serum (FBS), L-glutamine, streptomycin/penicillin solution, sodium bicarbonate, non-essential amino acids, sodium pyruvate were purchased from Sigma-Aldrich^®^.

Human malignant melanoma cells A-375 (CRL-1619™) and human amelanotic melanoma cells C32 (CRL-1585™) were purchased from American Type Culture Collection (ATCC, USA). Normal human HaCaT keratinocytes were obtained from The German Cancer Research Center (Deutsches Krebsforschungszentrum, DKFZ), where scientists successfully established a spontaneously immortalized cell line^30^.

### Methods

#### Liposomes preparation

**Fig. 2:**
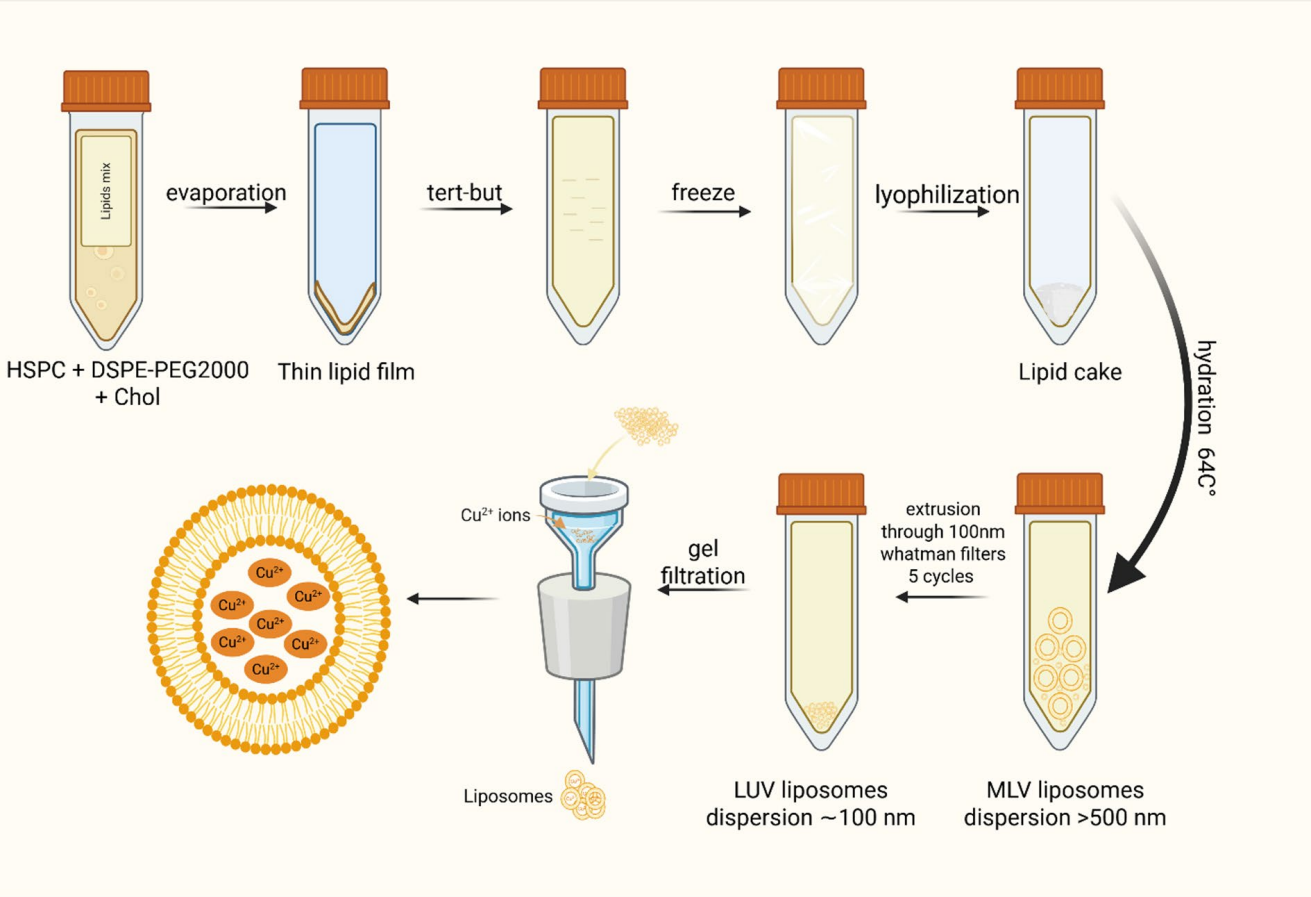
The scheme for the procedure of unloaded liposomes preparation. Created with BioRender.com (Science Suite Inc., Canada).

Liposomes were prepared using the film method^31^. Briefly, HSPC, DSPE-PEG2000, and Chol were dissolved in chloroform to obtain a stock solution of 10 mg·mL⁻¹. Chloroform lipid mixtures of HSPC: DSPE-PEG2000:Chol (55:5:40) were placed into glass probes to obtain a total of 40 mg of lipids. Then, chloroform was evaporated in a water bath (POLSONIC Palczynski Sp. J., Poland) under nitrogen gas for a few minutes, and the resulting thin lipid film was thoroughly dissolved in 2 ml of tert-butanol using the water bath. Dissolving a thin lipid film in tert-butanol allows even distribution of the molecules and the formation of a homogeneous mixture, which can then be frozen and lyophilized. Moreover, this solvent easily sublimates and does not disrupt lipid structures, thereby preserving the integrity and physicochemical properties of the resulting liposomes. After that, the tert-butanol solution of lipids was frozen in liquid nitrogen to ensure complete solidification. This rapid freezing step minimizes ice crystal formation and preserve liposomal integrity. No cryoprotectant was used. Then, frozen samples were transferred to the freeze dryer pre-cooled to − 40 °C and freeze-dried under vacuum (pressure of approximately 100 mTorr) overnight using a Savant Modulyo Freeze Dryer lyophilized (Thermo Fisher Scientific™, USA). The shelf temperature was maintained at − 40 °C for 2 h, then gradually increased to − 20 °C over 8 h. Following primary drying, the shelf temperature was gradually increased from − 20 °C to 25 °C over 6 h, and maintained at this temperature for 8 h. After lyophilization, the lipid cake was obtained and was hydrated with 2 ml of 150 mM copper (II) sulfate using the water bath for 5 min and vortexed (IKA^®^ Vortex 2, IKA-Werke GmbH & CO. KG, Germany). Probes were vortexed once for about a minute until the solution was well-mixed and homogeneous. Hydration was performed until the lipid cake was fully hydrated to form multilamellar vesicles (> 500 nm), referred to as MLV. The MLV liposome dispersion was extruded using a 10 ml thermobarrel extruder (Lipex^®^ Extruder, Evonik, Germany), through 100 nm Whatman^®^ Nuclepore polycarbonate filters (Whatman^®^ Nuclepore Track-Etched Membranes, Sigma-Aldrich^®^, USA) five times. After 5 cycles of extrusion at 64 °C, large unilamellar vesicle (LUV) liposomes were obtained. The liposomes were purified on a chromatography column packed with Sephadex^®^ G-50 Fine (Sigma-Aldrich^®^, USA) to remove metal ions that were not encapsulated in the hydrophilic interior of liposomes. They were then stored in Eppendorf^®^ tubes (Eppendorf^®^, Germany) for further assessment. The scheme for the procedure of unloaded liposome preparation is shown in Fig. 2.

**Fig. 3:**
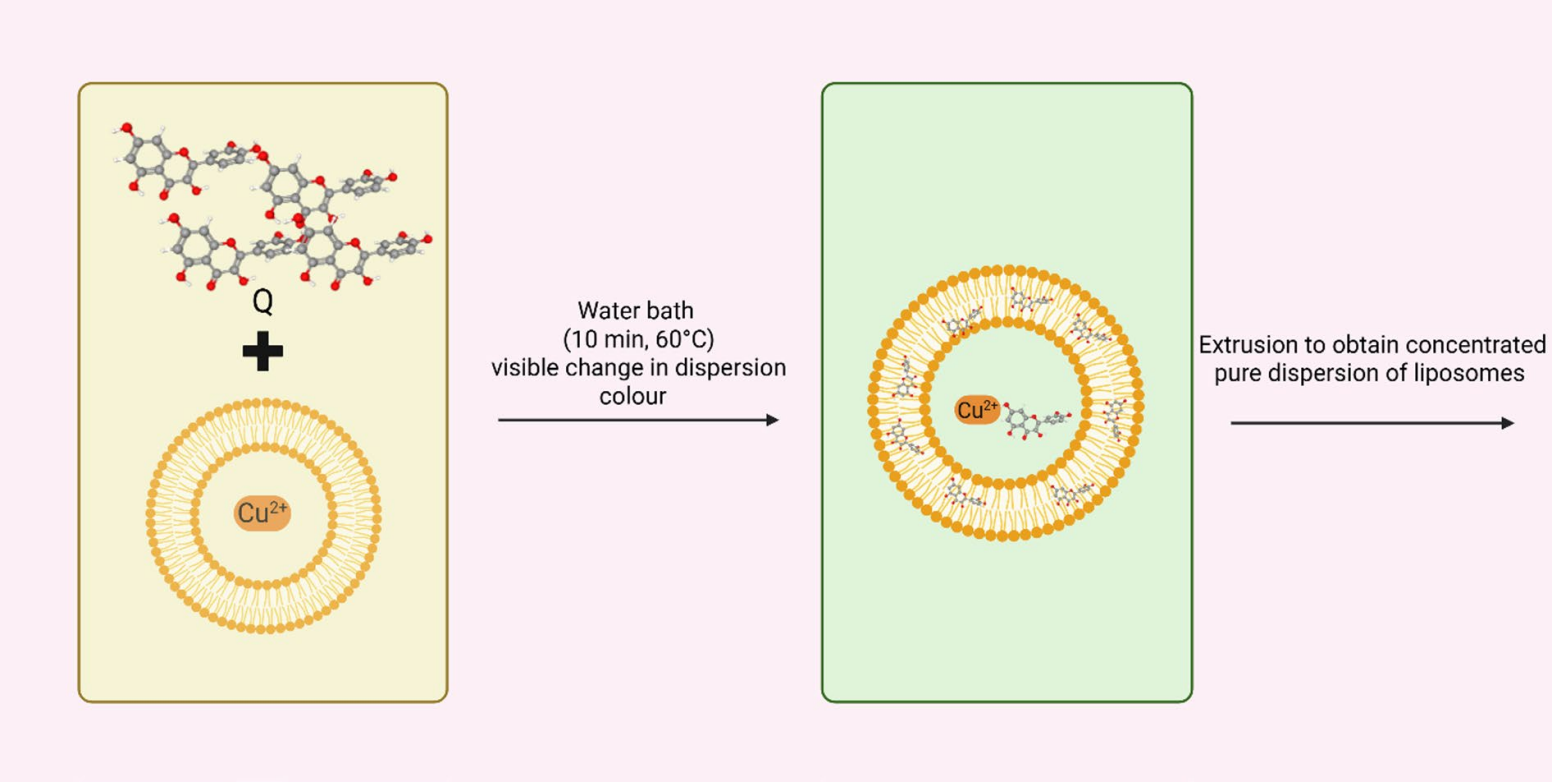
The procedure of Q encapsulation in liposomes. Created with BioRender.com (Science Suite Inc., Canada).

Total phospholipid content in the liposomes dispersion was determined by the Stewart method based on the formation of a chloroform-soluble complex between iron (III) thiocyanate and phospholipids^32^. For this purpose, 10 µl of liposome dispersion was transferred into a tube and brought up to 2 ml with chloroform. Then, 2 ml of Stewart’s reagent containing iron (III) thiocyanate was added and the mixture was shaken vigorously using a vortex for about 30 s. The contents of the tubes were centrifuged at 1050 rpm for 5 min (Sigma 3–18 K, Sigma-Aldrich^®^, USA). The lower phase of the solution was collected into a quartz cuvette, and its absorbance at 485 nm was measured using a Shimadzu^®^ TCC-240 A UV-Vis spectrophotometer (Shimadzu Corp^®^, Japan). After colorimetric determination of the phospholipid concentration, the initial liposome dispersion was diluted with 0.9% sodium chloride to a final phospholipid concentration of 10 mg·mL⁻¹ to adjust the osmolarity to physiological levels and ensure liposome stability. For encapsulation, Q was dissolved in DMSO to obtain a 10 mg·mL⁻¹ stock solution. The best encapsulation efficiency was obtained for a formulation containing an initial liposome dispersion of 10 mg·mL⁻¹ and a total DMSO proportion of about 20% v/v, incubated in the water bath set at 60 °C for 10 min. DMSO at concentrations of 20% v/v or higher can have membrane-disruptive effects, including increased bilayer fluidity. In our study, the formulation was exposed to this DMSO level during the incubation to enhance the permeation of Q molecules through the lipid bilayer to form complexes inside the aqueous core. The concentration of Q in dispersion before gel filtration was 0.5 mg·mL⁻¹. The final step was to separate the liposomal dispersion of Q from unencapsulated Q and residual DMSO by gel filtration using a minicolumn packed with Sephadex^®^ G-50 Fine. The scheme for the procedure of Q encapsulation in liposomes is shown in Fig. 3. Composition of the empty (L) and Q loaded (LQ) liposomes is shown in Table 1.

**Table 1:** Composition, ratio and code of the unloaded and loaded liposome formulations.

Formulation	Composition	Molar ratio	Code
Unloaded liposome	HSPC: DSPE-PEG2000:Chol	55:5:40	L
Loaded liposome	HSPC: DSPE-PEG2000:Chol + Q	55:5:40	LQ

#### Determination of liposomes mean particle size and size distribution

Mean particle size, size distribution, and polydispersity index (PDI) were measured using a Zetasizer Nano ZS (Malvern Instruments, UK). The polydispersity coefficient reflects the homogeneity of liposomes in the dispersion. According to the literature, if its value does not exceed 0.3, the dispersion of liposomes is considered homogeneous^33^. Measurements were performed in ultrapure Milli-Q^®^ water at room temperature (RT). Dynamic light scattering (DLS) measurement graphs were generated using the built-in averaging software to obtain a single representative peak for each sample.

#### Determination of encapsulation efficiency (EE%)

Encapsulation efficiency (EE%) was determined by measuring the concentration of Q in the final liposome dispersion after the sample was separated on a Sephadex^®^ G-50 Fine microcolumn. The concentration of Q encapsulated in liposomes was determined by solubilizing the liposomal membrane, for which 10 µl of liposome dispersion, 980 µl of methanol and 10 µL of EDTA solution were used. The use of EDTA was aimed at releasing Q from its complex with copper ions encapsulated in the hydrophilic part of the liposomes. The concentration of Q in the liposome dispersion was determined by photometric measurement at 365 nm using a Shimadzu^®^ TCC-240 A UV-Vis spectrophotometer.

The content of Q was calculated using a standard calibration curve prepared in the concentration range of 1–10 µg.mL-1. The calibration curve exhibited good linearity and was described by Eq. (1):

1 $$y = 0.0592x + 0.0354$$

where *y* represents absorbance and *x* is the Q concentration (µg.mL-1). The correlation coefficient (R) was 0.994, calculated as the square root of the determination coefficient (R² = 0.9881), indicating a strong positive linear relationship between absorbance and concentration (Figure S1, *Supplementary Material*). The encapsulation efficiency (EE) was based on the ratio of the Q amount determined by the colorimetric assay in the liposomes to the theoretical maximum amount. EE was calculated according to Eq. (2):

 Eq. (2)

2 $$\:\mathrm{E}\mathrm{E}=\frac{\mathrm{A}\mathrm{m}\mathrm{o}\mathrm{u}\mathrm{n}\mathrm{t}\:\mathrm{o}\mathrm{f}\:\mathrm{Q}\:\mathrm{e}\mathrm{n}\mathrm{c}\mathrm{a}\mathrm{p}\mathrm{s}\mathrm{u}\mathrm{l}\mathrm{a}\mathrm{t}\mathrm{e}\mathrm{d}\:\mathrm{i}\mathrm{n}\:\mathrm{l}\mathrm{i}\mathrm{p}\mathrm{o}\mathrm{s}\mathrm{o}\mathrm{m}\mathrm{e}\mathrm{s}\text{}}{\mathrm{T}\mathrm{h}\mathrm{e}\mathrm{o}\mathrm{r}\mathrm{e}\mathrm{t}\mathrm{i}\mathrm{c}\mathrm{a}\mathrm{l}\:\mathrm{m}\mathrm{a}\mathrm{x}\mathrm{i}\mathrm{m}\mathrm{u}\mathrm{m}\:\mathrm{a}\mathrm{m}\mathrm{o}\mathrm{u}\mathrm{n}\mathrm{t}\:\mathrm{o}\mathrm{f}\:\mathrm{Q}}\mathrm{x}\:100\mathrm{\%}$$

where the theoretical maximum amount of Q, corresponding to 100% encapsulation efficiency, was 0.5 mg·mL⁻¹.

#### Stability evaluation of liposomes

The stability of liposomal dispersion was evaluated by measuring the parameters (mean particle size and PDI) of both loaded and unloaded liposomes immediately after preparation (day 0) and after storage at 4 °C for 30 days. To determine these parameters, 100 µL of liposome dispersion was transferred to an Eppendorf^®^ tube and centrifuged at 19,000 × g for 2 min to separate precipitated quercetin crystals from the liposomal dispersion. After centrifugation, the liposomal Q in the supernatant was separated from any Q crystals that may have precipitated from the liposomes. For loaded liposomes, the concentration of Q was also determined. The concentration of Q was measured as described above and compared to the initial concentration on day 0, which was considered 100% of the encapsulated Q.

#### Cell culture studies

A-375 cells were cultured in DMEM supplemented with 10% FBS and 1% L-glutamine with streptomycin/penicillin solution. C32 cells were cultured in EMEM supplemented with 10% FBS, 1.5 g.L-1 sodium bicarbonate, 1 mM non-essential amino acids, 1 mM sodium pyruvate, and 1% L-glutamine with streptomycin/penicillin solution. HaCaT cells were cultured in DMEM supplemented with 10% FBS, and 1% L-glutamine with streptomycin/penicillin solution. Cell cultures were maintained at 37 ℃ in a 5% CO_2_ humidified atmosphere (HeraCell 150i, Thermo Fisher Scientific). The media were changed twice a week. Cells were trypsinized with 0.25% Trypsin-EDTA solution when confluence did not exceed 70%.

#### SRB cell viability assay

The cytotoxicity of Q was evaluated using the SRB assay [30]. The cells were seeded onto 96-well plates at a density of 6 × 10^3^ cells per well for HaCaT, 3 × 10^3^ cells per well for A-375 and 2 × 10^3^ cells per well for C32, and incubated for 24 h prior to the experiment. The following day, 100 µL of the Q or liposomal Q was added to the media to obtain a range of concentrations: 2, 5, 10, 20, 25, 50, 100, 150 and 200 µM. Control cells were treated with complete media only and cells treated with medium containing liposomes without Q at the highest concentration were included as a solvent control. At the end of the incubation period, 50 µL of 50% cold TCA was added to each well and incubated for 1 h at 4 °C. The plate was then rinsed with water five times, dried, and 50 µL of 0.4% solution of SRB in 1% acetic acid was added to each well. After 30 min of incubation at RT, the plate was rinsed five times with 1% acetic acid and dried. 150 µL of 10 mM TRIS-HCl solution was added to each well and the plates were gently shaken for 10 min. The absorbance reading was conducted at 540 nm wavelength with the use of microplate reader (ELx 800, BIO-TEK Instruments, USA). In the SRB assay, which is a colorimetric test, the value of absorbance is proportional to cell viability, determined according to Eq. (3):

3 $${\rm V_{\%} = [(As - Am)/ (Ac - Am)] \times 100\%}$$

where: A_s_ – absorbance of the test sample, A_m_ – absorbance of the medium, A_c_ – absorbance of the control sample. Cell viability (V_%_) was expressed as a percentage of cells in the test sample relative to the control, which consisted of cells cultured in complete medium^34^. SRB assays were performed in three independent replicates.

### Statistical analysis

All statistical analyses were performed using Prism 10.0 (GraphPad Software Inc., USA). Normality of distribution was analyzed with Shapiro-Wilk test^35^. Statistical analyses were made with a one-way analysis of variance (ANOVA) test followed by Tukey’s multiple comparisons test for free Q. Statistical analyses for liposomal Q were made with a Kruskal-Wallis one-way analysis of variance by ranks and Dunn’s test^36^. A p-value of ≤ 0.05 was considered statistically significant. N refers to the sample size, and data are presented as the mean ± SD, which indicates the variability within the data.

## Results

### Characteristics of Q-loaded liposomes

Poor bioavailability of Q is related to its low water solubility, which is a major limitation in the development of effective drugs^37^. Therefore, a nanocarrier for the potential delivery of this compound was developed. Liposomes were prepared using the film method. The encapsulation efficiency (EE%) of Q incubated at 60 ℃ with liposomes hydrated with 150 mM copper(II) sulfate was measured. The visual appearance of the liposomes in the solution with Q before incubation was yellow, whereas after incubation it became green-colored and opalescent. The color of the liposome dispersion with encapsulated Q is due to the complex it forms with sulfate ions. Empty liposomes were also opalescent but the color was milky, as they did not contain encapsulated Q.

The parameters of liposomes were determined at three stages of their preparation: unloaded liposomes before extrusion, unloaded liposomes after extrusion, and loaded liposomes after encapsulation of Q. After hydration of the dry lipid cake, a heterogeneous population of MLV liposomes with a mean particle size of 1007 ± 101 nm was formed. After passing the liposome dispersion through a polycarbonate filter with a pore diameter of 100 nm during the extrusion process, LUV liposomes with an average size of about 100 nm were formed. The size and homogeneity of the liposomal dispersion were evaluated using the Malvern NanoZS Dynamic Light Scattering (DLS) system. The EE% for encapsulation of Q was 61%. Figure S2 (*Supplementary Material*) shows the DLS measurement graphs for the liposomal dispersion and Table 2 presents the results of the measurements. Data are presented as mean values ± SD from three independent measurements.

**Table 2:** Liposome Characterization.

Sample	Before extrusion	After extrusion	After encapsulation of Q
**Mean Particle Size and Size Distribution [nm]**	1007 ± 101	131.1 ± 26.3	169.9 ± 33
**PDI**	0.631	0.046	0.204
**Encapsulation** **efficiency [%]**	-	-	61

As shown in Table 2, the DLS results indicated that the obtained liposomal dispersion before extrusion had a mean particle size of 1007 ± 101 nm and a PDI of 0.631, suggesting that, at this stage, a population of MLV liposomes was obtained. PDI values exceeding 0.3 indicate that this liposomal dispersion was not homogeneous. After extrusion, the average liposome size for empty liposomes used for encapsulation was 131.1 ± 26.3 nm, and the PDI fell below 0.3 indicating that this formulation had a homogeneous size distribution. After encapsulation of Q in the liposomes, the PDI increased, but its value remained below 0.3. The average size of the liposomes increased to 169.9 ± 33 nm, which could be due to fusion of liposome membranes into larger vesicles following the addition of Q dissolved in DMSO and incubation at an elevated temperature. Empty liposomes, composed of neutral lipids HSPC and cholesterol with DSPE-PEG2000 providing surface PEGylation, exhibited a near-neutral surface charge with a zeta potential of approximately − 1 mV. Although the zeta potential of Q-loaded liposomes was not directly measured in this study, previous work using the same lipid composition and active loading approach with another flavonoid, aloe-emodin, showed that encapsulation via a copper ion gradient did not significantly affect the liposome zeta potential. Based on these results, Q encapsulation is also expected to have minimal impact on surface charge, indicating that the liposomal surface remains near-neutral after drug loading. In this study, neutral liposomes were chosen to minimize nonspecific interactions with serum proteins, reduce charge-related cytotoxicity, and better mimic the near-neutral charge of physiological membranes.

### Stability studies of liposomes

A stability assay was conducted for LQ liposomes to determine changes in size and homogeneity of the liposomal dispersion after 30 days of storage in 4 ℃. The samples were sealed tightly in Eppendorf^®^ tubes and protected from external factors using sealing film (Parafilm^®^ M Sealing Film, Sigma-Aldrich, USA). To assess this, 100 µL of the liposomes were taken from the sample and centrifuged at 19,000 × g for 2 min. The liposomal Q in the supernatant was then separated from the Q precipitate that had leaked from the aqueous phase of the liposomes. The concentration of the remaining encapsulated Q in the liposomes was also spectrophotometrically determined as described previously (Sect. 2.2.3) and compared to the baseline Q concentration on day 0, which was determined to be 100% of the encapsulated compound. After one month, only a small precipitate of Q was observed at the bottom of the Eppendorf^®^ tube. The spectrophotometric measurements of the concentration showed a loss of about 16% of the encapsulated Q: the concentration of encapsulated Q decreased from 0.305 mg·mL⁻¹ on day 0 to 0.254 mg·mL⁻¹ after 30 days. The stability test showed that the average size of the liposomes increased from 169.9 ± 33 nm to 187.5 ± 46.9 nm after one month, indicating that after this time the liposome membranes began to fuse to form larger vesicles. The PDI also increased from 0.204 to 0.317, but the value still remained low, suggesting that the initial homogeneity was retained. Graphs showing the changes in vesicle size distribution for each formulation after one month are shown in Figure S3 (*Supplementary Material*) and the results of the experiment are also presented in Table 3. Data are presented as mean values ± SD from three independent measurements.

**Table 3:** Parameters of liposomal dispersion measured after preparation (day 0) and after 30 days.

Parameters of liposomes (day 0)			Parameters of liposomes (day 30)		
**Mean Particle Size and Size Distribution [nm]**	**PDI**	**Q concentration [mg·mL**^-1^]	**Mean Particle Size and Size Distribution [nm]**	**PDI**	**Q concentration [mg·mL**^-1^]
169.9 ± 33	0.204	0.305	187.5 ± 46.9	0.317	0.254

### Cytotoxic activity studies

**Fig. 4:**
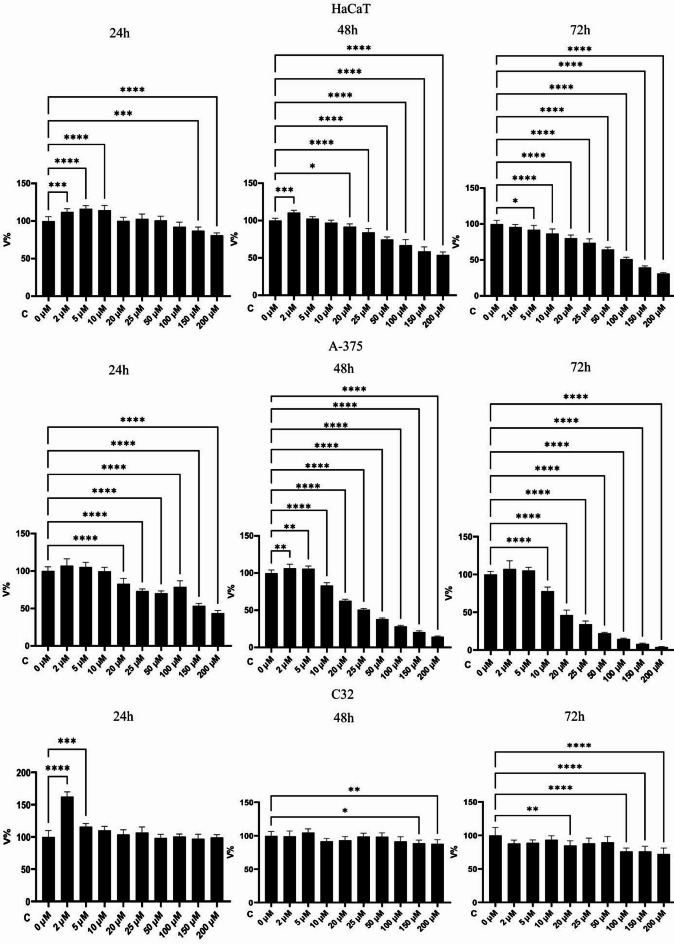
Cytotoxity of free Q. The SRB assay was performed on the immortalized human keratinocytes HaCaT, human melanoma A-375 and human amelanotic melanoma C-32 cells at a wide range of concentrations of free Q. Control (0 µM) with medium was included in the experiment. One-way ANOVA followed by Tukey’s multiple comparisons test; V% – percentage of cell viability; significant p-value ≤ 0.05; * *p* ≤ 0.05, ** *p* ≤ 0.01, *** *p* ≤ 0.001, **** *p* ≤ 0.0001.

**Fig. 5:**
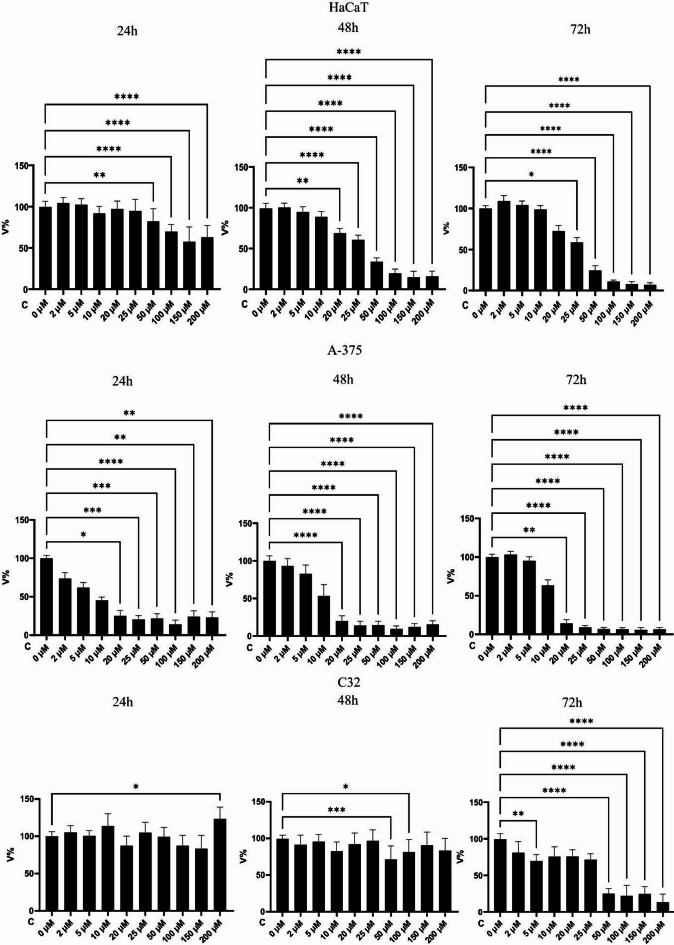
Cytotoxity of liposomal Q. The SRB assay was performed on the immortalized human keratinocytes HaCaT, human melanoma A-375 and human amelanotic melanoma C-32 cells at a wide range of concentrations of liposomal Q. Control (0 µM) with medium was included in the experiment. Kruskal-Wallis one-way analysis of variance by ranks and Dunn’s test^36^; V% – percentage of cell viability; significant p-value ≤ 0.05; * *p* ≤ 0.05, ** *p* ≤ 0.01, *** *p* ≤ 0.001, **** *p* ≤ 0.0001.

To evaluate the anticancer potential of Q and LQ liposomes, their in vitro cytotoxicity was investigated against two human melanoma cell lines (A-375 and C32) in comparison to normal human keratinocytes (HaCaT). During the experiments, cells were incubated for 24, 48 and 72 h with liposomal formulations containing encapsulated Q at concentrations ranging from 2 to 200 µM. Non-loaded liposomes at the highest concentration were used as a solvent control and untreated cells served as a negative control. The experimental outcome was determined using the SRB assay. The results presented in Figs. 4 and 5 and S4-S6 (*Supplementary Material*) showed a dose- and time-dependent anticancer effect of Q, both as a free compound and when encapsulated in liposomes.

In the case of free Q, after 24 h, a statistically significant increase in cell survival was observed in HaCaT cells at lower concentrations of Q (2–10 µM) (*p* ≤ 0.001), whereas a significant decrease in cell survival was seen only at concentrations above 150 µM (*p* ≤ 0.001). After 48 h, free Q showed cytotoxic effects even at much lower concentrations, starting from 20 µM and above. After 72 h of incubation, this relationship became even more pronounced. For the A-375 cells, the cytotoxic effect of Q was observed at a concentration of 20 µM after 24 h, while after 48 and 72 h the cytotoxic effect was noticeable even at a concentration of 10 µM.

The amelanotic melanoma cells (C32) showed the greatest resistance to treatment with free Q. A statistically significant decrease in cell survival was observed only after 48 h for concentrations above 150 µM (*p* ≤ 0.05), and after 72 h for concentrations above 100 µM (*p* ≤ 0.0001) (Fig. 4, Figure S4; *Supplementary Material*).

The results obtained for HaCaT cells suggest that the survival rate of cells treated with liposomal Q at concentrations of 2 µM and 5 µM was comparable to, or even higher than that of control cells not treated with the compounds. This supports the hypothesis, based on the literature, that Q at low concentrations has a protective effect on normal cells and promotes their proliferation^38^. After 24 h, a statistically significant decrease in viability was observed for liposomal Q at concentrations of 50 (*p* ≤ 0.01), 100, 150, and 200 µM (*p* ≤ 0.0001). After 48 h, a significant difference in viability was also observed for concentrations of 20 (*p* ≤ 0.01) and 25 µM (*p* ≤ 0.0001). After 72 h, this dependence of cell survival on the concentration of liposomal Q became even more pronounced. In the case of the melanoma cell line A-375, cell survival significantly decreased at a concentration of 20 µM (*p* ≤ 0.05), and after 48 h, survival dropped to approximately 50% at a concentration of 10 µM. The results for the C32 melanoma cells after 24 h showed that this line was much less sensitive to the effect of Q encapsulated in liposomes. Even the highest concentrations of Q did not significantly reduce cell viability. Only after 48 h did the effect of liposomal Q become more noticeable, and from a concentration of 50 µM upwards, cell survival decreased significantly (*p* ≤ 0.001). After 72 h, the effect of Q at concentrations above 50 µM was even more pronounced (Fig. 5, Figure S5; *Supplementary Material*).

Comparing the effect of free Q with liposomal Q in the case of HaCaT cells, no statistically significant differences were observed. Interestingly, in the case of A-375 cells, Q encapsulated in liposomes exhibited a significantly stronger cytotoxic effect than the free form in the concentration range of 10–100 µM after 24 h (*p* ≤ 0.05). However, after longer incubation times, this difference was no longer significant. For C32 cells, a significant difference between the two forms of Q was observed only after 72 h, for concentrations above 50 µM, with liposomal Q showing greater efficacy (*p* ≤ 0.01) (Figure S6; *Supplementary Material*).

## Discussion

Q, according to numerous scientific studies^5, 16, 24, 25, 26^, affects the process of carcinogenesis and induces apoptosis in cancer cells, making it a potential candidate for anticancer therapy. However, due to its chemical structure, this compound is poorly absorbed in the body and has relatively low bioavailability, which limits its potential use in the therapy. The research conducted in this study aimed to utilize liposome technology to encapsulate Q, thereby enhancing its bioavailability which is limited by poor water solubility and rapid metabolism. The liposomal form of Q is believed to exhibit improved bioavailability compared to its free form, making it a promising candidate for anticancer therapy. Q’s ability to bind copper has previously been utilized in the development of liposomes containing this flavonoid^39^. Researchers obtained a stable liposomal Q formulation suitable for intravenous administration, which showed no apparent toxicity when administered at doses of 50 mg·kg^− 1^ in mice. They postulated that this novel Q formulation could also be useful for anticancer therapy^18^. The formulation we tested was obtained in a similar manner. However, it differed in the composition of the lipid bilayer, which consisted of a mixture of HSPC: DSPE-PEG2000:Chol (55:5:40). Furthermore, the cytotoxic effect of this formulation was evaluated for the first time through in vitro tests on melanoma cells A-375 and C32. In this work, the parameters of Q-loaded liposomes with the appropriate size and homogeneity for intravenous applications have been developed and evaluated. In vitro tests demonstrated the efficacy of liposomal Q in treating melanotic melanoma, positioning these liposomes as a potential anticancer drug for further studies or in vivo experiments. In vitro results showed that Q, at lower concentrations, did not negatively affect the survival of normal cells, and even promoted their proliferation. However, higher concentrations of Q significantly reduced cell survival (*p* ≤ 0.0001). Based on these findings, it can be concluded that the cell lines exhibited different sensitivities to Q. The A-375 melanoma cell line was found to be the most sensitive, with liposomal Q significantly reducing cell viability after just 24 h, already at a concentration of 20 µM (*p* ≤ 0.05). Although the mechanism of Q’s action on melanoma cells has not been fully investigated, one study demonstrated that its antitumor activity is at least partially mediated through the inhibition of signal transducer and activator of transcription 3 (STAT3) signaling, resulting in suppressed A-375 tumor growth in a xenograft mouse model^40^. Moreover, another study on the same cell line showed that Q can modulate Wnt/β-catenin signaling and apoptotic pathways^41^. Another likely mechanism of action of Q is related to the expression of tyrosinase, which has been shown to increase in tumors arising from melanocytes. The oxidation of Q by this enzyme can lead to a enhancement of its pro-oxidant effects and enhance apoptosis in melanoma cells ^42^. Comparing the results obtained for the two melanoma cell lines, it can be concluded that the C32 amelanotic melanoma cells are much more resistant to Q than the A-375 melanotic melanoma cells. Even high concentrations of Q did not reduce cell survival after 24 h, and only concentrations above 50 µM significantly affected C32 cells after prolonged incubation (*p* ≤ 0.0001). This difference in cytotoxicity may be explained by the limited tyrosinase activity in amelanotic melanoma due to aberrant retention of tyrosinase in the endoplasmic reticulum, which leads to increased degradation in the proteasomes of amelanotic melanoma cells^43^. Furthermore, Q demonstrates the ability to inhibit the activity of P-glycoprotein (P-gp), whose overexpression is directly associated with the development of multidrug resistance (MDR) in cancer cells. This is the main cause of chemotherapy failure, as P-gp leads to the expulsion of drug molecules from the cell. Previous studies have shown that a likely mechanism of Q’s action is the modulation of P-gp through the inhibition of signal transduction from the nucleotide-binding domain to the transmembrane domain ^44^. Another study showed that Q can reverse MDR by significantly inhibiting the metabolism of D-glutamine and D-glutamate. The likely mechanism of this phenomenon is that Q reduces the expression of the glutamine transporter from the solute carrier family 1, member 5 (SLC1A5). Scientists have shown that Q is an inhibitor of SLC1A5, and its inhibition resulted in a reduction in cell proliferation in several cancer types, including melanoma^45, 46^. The effect of Q observed in C32 cells may suggest that it initially acted as a potential chemosensitizer, which would explain why the cytotoxic effect was only observed after prolonged exposure. It is worth noting that Q-loaded liposomes exhibit similar anticancer activity compared to the unencapsulated compound. Although the liposomal formulation did not show a significant advantage over free quercetin in the in vitro assays, it is important to notice that liposomal delivery systems are generally expected to provide significant advantages in vivo. These benefits include enhanced bioavailability, increased stability and more favorable pharmacokinetic properties, which may ultimately improve the therapeutic performance of quercetin. Moreover, as known from clinically used liposomal drugs such as liposomal doxorubicin, reduced apparent in vitro potency does not preclude improved in vivo efficacy. In addition to the potential use of the liposomal form of Q in monotherapy, its application in combination therapy is also promising, as Q may work synergistically with other compounds, such as cisplatin or 5-fluorouracil, to enhance their anticancer effects ^47, 48^. From the perspective of using the liposomal form of Q in melanoma therapy, it is also important to note that tyrosinase overexpression in cells promotes an ATM-dependent p53 phosphorylation by Q treatment and sensitizes melanoma cells to DTIC, but also to temozolomide (TMZ), the more commonly used analog of DTIC-related oral agents^26, 49^. Moreover, the surface of the liposomes could be further modified to enable targeting of cancer cells^50, 51, 52^. One limitation associated with the use of drug-loaded liposomes is their relatively low stability, as liposomes can aggregate and fuse into larger structures - a process that is particularly unfavorable for their use in anticancer therapy. Experiments with liposomes uncovered that the smallest liposomes are the most fusogenic^53^. Other challenges include potential drug leakage and uptake of the liposomes by reticuloendothelial system (RES), leading to their removal from the body before reaching the therapeutic target^54, 55, 56^.

This research presents results from preliminary studies. While the findings are promising, further research involving larger datasets and more refined methodologies is required to draw conclusions. In addition, thorough characterization of long-term stability remains a critical requirement for advancing pharmaceutical development and meeting regulatory standards.

## Conclusions

Summarizing the presented data, the use of the liposomal form of Q offers advantages over its free form, as it addresses the issues of poor solubility and bioavailability. However, liposomal carriers are not without their drawbacks. As studies have shown, one potential issue that needs further investigation is the stability of liposomes. The membranes of liposomes in dispersion can fuse and aggregate, leading to changes in their size over time and to the premature release of the drug from their interior, which is an undesirable phenomenon. Film technique has been studied in order to obtain the optimum formulation consisting of HSPC: DSPE-PEG2000:Chol (55:5:40) for encapsulating the drug in liposomes to achieve the highest possible encapsulation efficiency while considering its chemical structure. Based on one-month stability studies, this formulation was stable for this period of time while stored at 4 °C. However, longer-term stability studies under various storage conditions are necessary to determine whether the formulation is robust and shelf-stable for real-world applications. An important consideration, in addition to developing the liposomal form of the drug, is that different types of cancer may have varying sensitivities to therapy, which is one of the primary reasons for treatment failure. In conclusion, our studies have shown that Q encapsulated in liposomes may have potential applications in anticancer therapy, as it exhibits cytotoxic effects on cancer cells without negatively affecting normal cells. Moreover, its use in combination with currently used chemotherapy, as this compound may also synergistically enhance the anticancer efficacy of other drugs.

In conclusion, this research provides insights based on preliminary studies. The results serve as a foundation for further investigations rather than definitive evidence of real-world therapeutic use. Future work will focus on comprehensive stability assessments, including evaluation of lyophilized or otherwise optimized liposomal systems to improve product robustness, as well as detailed analyses of the molecular mechanisms underlying the inhibition of malignancy.

## Supplementary Information

Below is the link to the electronic supplementary material.

Supplementary Material 1

## Data Availability

All the data supporting the findings of this study are available from the corresponding author on reasonable request.

## Abbreviations

Abbreviation: Description
ANOVA: Analysis of variance
ATCC: American Type Culture Collection
BRAF: B-Raf proto-oncogene, serine/threonine kinase
Chol: Cholesterol
DKFZ: The German Cancer Research Center (Deutsches Krebsforschungszentrum)
DLS: Dynamic Light Scattering
DMEM: Dulbecco’s Modified Eagle Medium
DMSO: Dimethylsulfoxide
DSPE-PEG2000: N-(carbonyl-methoxypolyethylene glycol-2000)-1,2-distearoyl-sn-glycero-3-phosphoethanolamine 2000
DTIC: Dacarbazine
EDTA: Ethylenediaminetetraacetic acid
EE: Encapsulation Efficiency
EMEM: Minimum Essential Medium Eagle With Earle′s salts
ERK: Mitogen-activated protein kinase 1
FBS: Fetal Bovine Serum
FDA: Food and Drug Administration
HSPC: Hydrogenated Soy Phosphatidylcholine
LQ: Quercetin Loaded Liposomes
LUV: Large Unilamellar Vesicle
MAPK: Mitogen-Activated Protein Kinase
MDR: multidrug resistance
MEK: Mitogen-Activated Protein Kinase
MLV: Multilamellar Vesicles
PBS: Phosphate Buffered Saline
PDI: Polydispersity Index
P-gp: P-glycoprotein
Q: Quercetin
RES: Reticuloendothelial System
RT: Room Temperature
SLC1A5: Glutamine transporter from the solute carrier family 1, member 5
SRB: Sulforhodamine B
STAT3: Signal Transducer and Activator of Transcription 3
TCA: Trichloroacetic acid
TKI: Tyrosine Kinase Inhibitors
TMZ: Temozolomide
