# Supplementary material for: Development of new liposomal formulations of quercetin – in vitro study
Source: Sci Rep. 2026 Apr 18;16:17953. doi: 10.1038/s41598-026-48068-3 (PMC13250069; doi:10.1038/s41598-026-48068-3)
Supplement: Supplementary file 1 — Supplementary Material 1 [file 41598_2026_48068_MOESM1_ESM.docx]

**SUPPLEMENTARY MATERIAL**

**Development of new liposomal formulations of quercetin – *in vitro* study**

Klaudia Krawczyńska^1*^, Agnieszka Rusak^1^, Tomasz Górnicki^1^, Monika Mrozowska^1^, Piotr Dzięgiel^1,2^, Jerzy Gubernator^3^

*^1^ Division of Histology and Embryology, Department of Human Morphology and Embryology, Wroclaw Medical University, Tytusa Chalubinskiego 6a St., 50-368 Wroclaw, Poland*

*^2^ Department of Human Biology, Faculty of Physiotherapy, Wroclaw University of Health and Sport Sciences, Paderewskiego 35 Avenue, 51-612 Wrocław, Poland*

*^3^ Department of Lipids and Liposomes, Faculty of Biotechnology, University of Wrocław, Joliot-Curie 14a St., 50-383 Wroclaw, Poland*

*^*^ Corresponding author: Klaudia Krawczyńska (klaudia.krawczynska@umw.edu.pl)*

**Figure S1. The standard curve for quercetin**


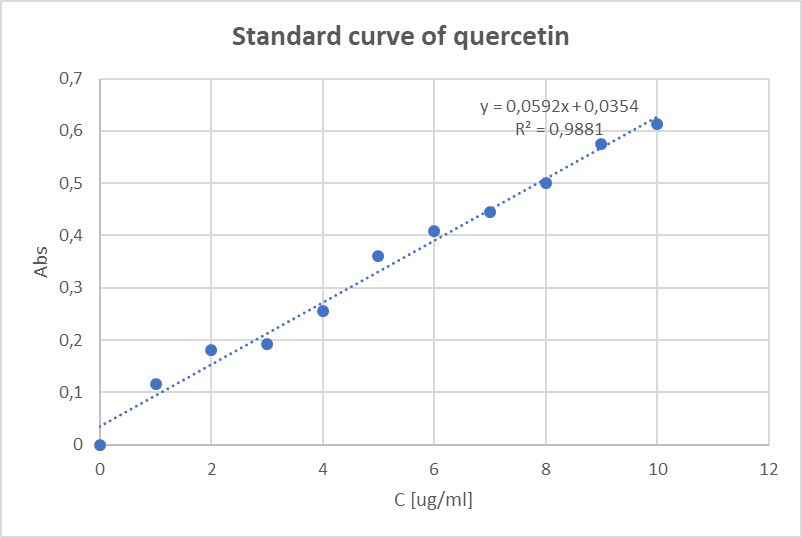


**Figure S2. DLS measurement results for each of the nanoparticles, with size [nm] and PDI values** (A) unloaded liposomes before extrusion, (B) unloaded liposomes after extrusion, (C) loaded liposomes after encapsulation of quercetin

A

*
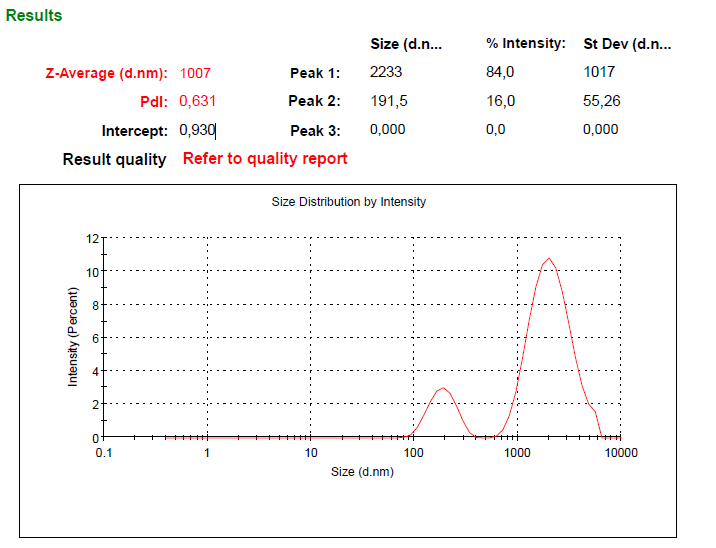
*

B


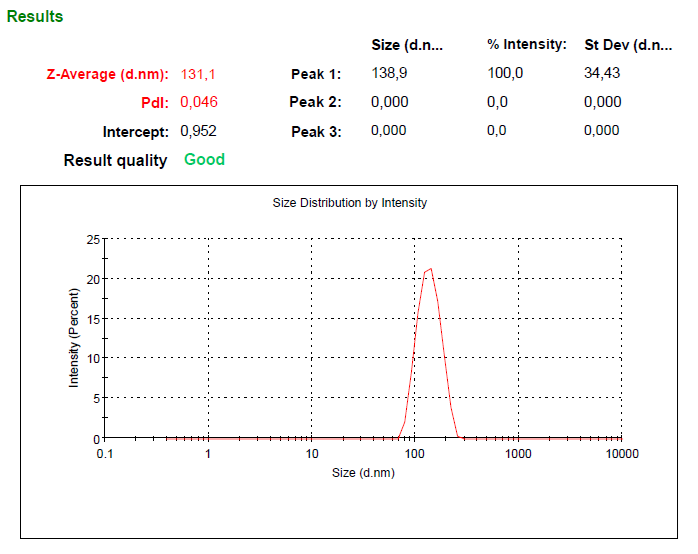


C


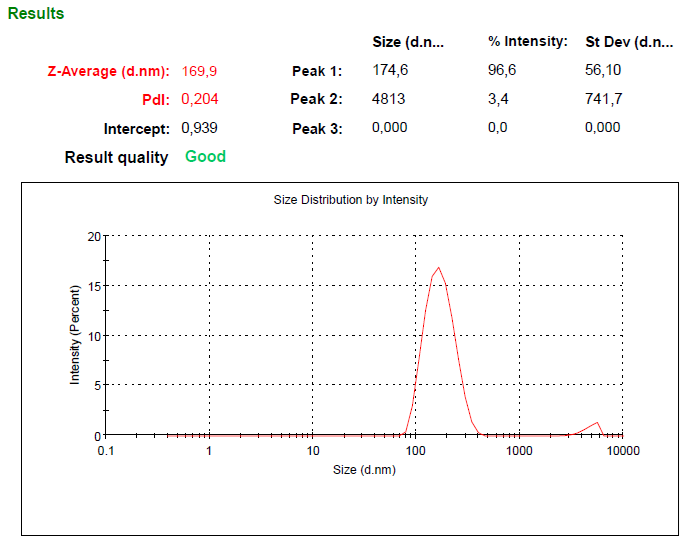


**Figure S3. DLS measurements results for each of the nanoparticles, with size [nm] and PDI values shown** (A) Q-loaded liposomes on day 0 (B) Q-loaded liposomes on day 30

A


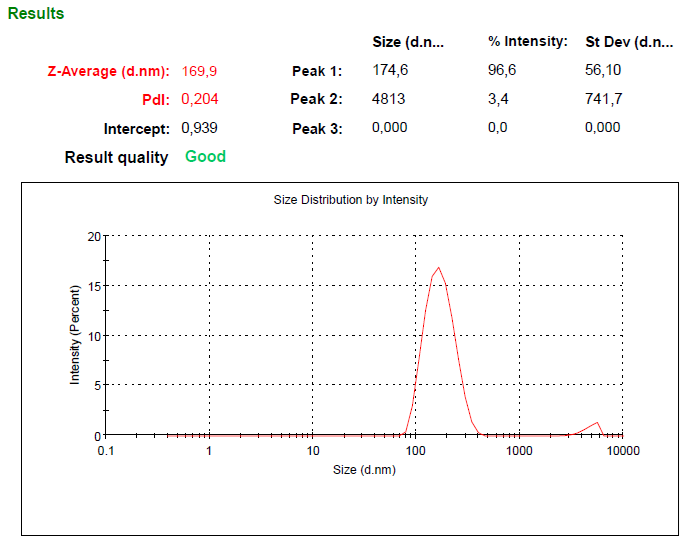


B


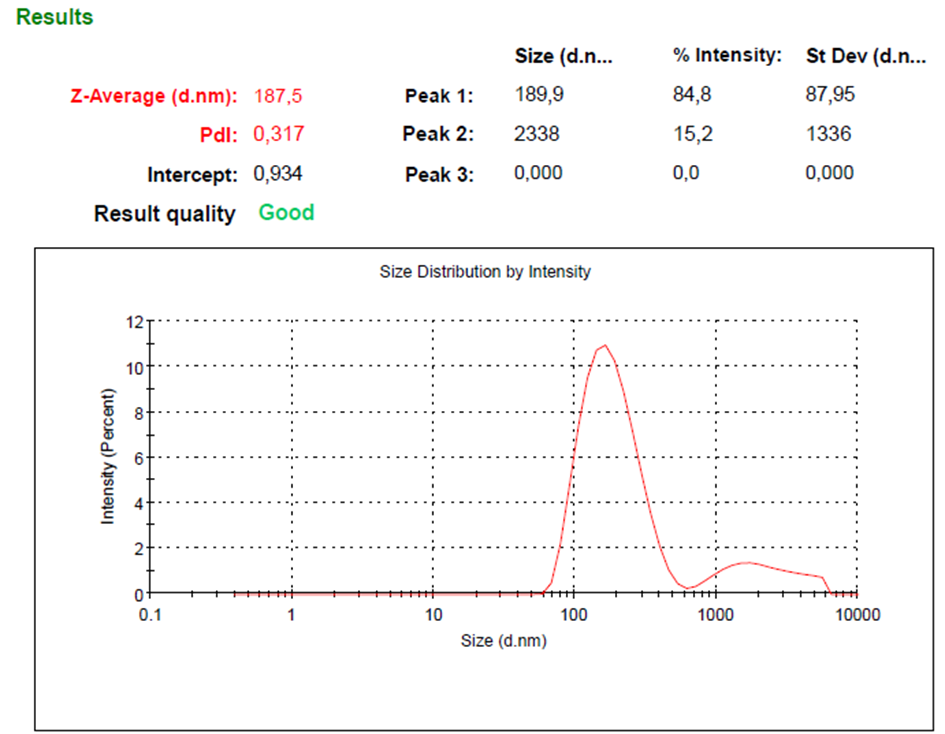


**Figure S4.** **Analysis of changes in cell viability between different incubation times for the same concentration of free quercetin.**

The SRB assay was performed on the immortalized human keratinocytes HaCaT, human melanoma A-375 and human amelanotic melanoma C-32 at a wide range of concentrations of free quercetin. One-way ANOVA followed by Tukey’s multiple comparisons test was performed. V% – percentage of cell viability; significant p-value ≤0.05; ns – not significant, * p≤0.05, ** p≤0.01, *** p≤0.001, **** p≤0.0001

**HaCaT C=2 μM**

**HaCaT C=5 μM**

**HaCaT C=10 μM**

**HaCaT C=20 μM**

**HaCaT C=25 μM**

**HaCaT C=100 μM**

**HaCaT C=150 μM**

**HaCaT C=200 μM**

**A-375 C=2 μM**

**A-375 C=5 μM**

**A-375 C=10 μM**

**A-375 C=20 μM**

**A-375 C=25 μM**

**A-375 C=50 μM**

**A-375 C=100 μM**

**A-375 C=150 μM**

**A-375 C=200 μM**

**C32 C=2 μM**

**C32 C=5 μM**

**C32 C=10 μM**

**C32 C=20 μM**

**C32 C=25 μM**

**C32 C=50 μM**

**C32 C=100 μM**

**C32 C=150 μM**

**C32 C=200 μM**

**Figure S5. Analysis of changes in cell viability between different incubation times for the same concentration of liposomal quercetin.**

The SRB assay was performed on the immortalized human keratinocytes HaCaT, human melanoma A-375 and human amelanotic melanoma C-32 at a wide range of concentrations of liposomal quercetin. Kruskal-Wallis one-way analysis of variance by ranks and Dunn’s test was performed. V% – percentage of cell viability; significant p-value ≤0.05; ns – not significant, * p≤0.05, ** p≤0.01, *** p≤0.001, **** p≤0.0001

**HaCaT C=2 μM**

**HaCaT C=5 μM**

**HaCaT C=10 μM**

**HaCaT C=20 μM**

**HaCaT C=25 μM**

**HaCaT C=50 μM**

**HaCaT C=100 μM**

**HaCaT C=150 μM**

**HaCaT C=200 μM**

**A-375 C=2 μM**

**A-375 C=5 μM**

**A-375 C=10 μM**

**A-375 C=20 μM**

**A-375 C=25 μM**

**A-375 C=50 μM**

**A-375 C=100 μM**

**A-375 C=150 μM**

**A-375 C=200 μM**

**C32 C=2 μM**

**C32 C=5 μM**

**C32 C=10 μM**

**C32 C=20 μM**

**C32 C=25 μM**

**C32 C=50 μM**

**C32 C=100 μM**

**C32 C=150 μM**

**C32 C=200 μM**

**Figure S6. Analysis of changes in cell viability between liposomal and free quercetin.**

The SRB assay was performed on the immortalized human keratinocytes HaCaT, human melanoma A-375 and human amelanotic melanoma C-32 at a wide range of concentrations of free quercetin. Kruskal-Wallis one-way analysis of variance by ranks and Dunn’s test were performed. V% – percentage of cell viability; significant p-value ≤0.05; ns – not significant, * p≤0.05, ** p≤0.01, *** p≤0.001, **** p≤0.000; w- free quercetin, l- liposomal quercetin

**HaCaT 24h**

**HaCaT 48h**

**HaCaT 72h**

**A-375 24h**

**A-375 48h**

**A-375 72h**

**C32 24h**

**C32 48h**

**C32 72h**
